# The deterioration of mental health among healthcare workers during the COVID-19 outbreak: A population-based cohort study of workers in Japan

**DOI:** 10.5271/sjweh.3922

**Published:** 2020-10-30

**Authors:** Natsu Sasaki, Reiko Kuroda, Kanami Tsuno, Norito Kawakami

**Affiliations:** 1 Department of Mental Health, Graduate School of Medicine, The University of Tokyo, Tokyo, Japan; 2 Division for Environment, Health and Safety, The University of Tokyo, Tokyo, Japan; 3 School of Health Innovation, Kanagawa University of Human Services, Kanagawa, Japan

**Keywords:** anxiety, depression, nurse, physician, SARS-Cov-2

## Abstract

**Objectives:**

This study compared the longitudinal change in the mental health of healthcare and non-healthcare workers during two months of the COVID-19 outbreak in Japan.

**Methods:**

Data were derived from a prospective online cohort study of 1448 full-time employees in Japan. Participants were surveyed at baseline from 19–22 March 2020 (T1) and at follow-up from 22–26 May 2020 (T2). A self-administered online questionnaire was used to assess participants’ fear and worry of COVID-19, psychological distress, and physical symptoms at T1 and T2. A series of generalized linear models were created to assess changes in outcomes between healthcare and non-healthcare workers. Demographic variables (ie, sex, age, marital status, child[ren], education, and residential area) were included in the models as covariates.

**Results:**

A total of 1032 participants completed the follow-up questionnaire at T2 (follow-up rate, 72.6%). After excluding unemployed respondents (N=17), the final sample comprised 1015 full-time employees (111 healthcare and 904 non-healthcare workers). After adjusting for the covariates, psychological distress (and subscales of fatigue, anxiety, and depression) as well as fear and worry of COVID-19 increased statistically significantly more among healthcare than non-healthcare workers from T1 to T2.

**Conclusions:**

Psychological distress, together with fear and worry of COVID-19, increased more among healthcare compared to non-healthcare workers during the COVID-19 outbreak. The study confirmed that healthcare workers are an important target for mental healthcare during the COVID-19 outbreak.

Poor mental health was reported among healthcare workers (HCW) during the COVID-19 outbreak of 2019–2020 (1–5). Recent systematic reviews of previous studies reported that HCW showed moderate-to-high levels of psychiatric symptoms such as anxiety, depression, insomnia (6), and acute and posttraumatic stress symptoms (7, 8) during the COVID-19 outbreak. Front- and second-line HCW, including not only physicians and nurses but also other allied healthcare professionals, manifested psychiatric symptoms (2, 9). These studies’ findings suggest that HCW are major targets of mental healthcare (1, 6).

However, evidence showing that HCW developed poorer mental health compared to non-HCW in the COVID-19 outbreak is still limited. A study in China reported that HCW and non-HCW experienced similar levels of anxiety and depression, while HCW showed a poorer quality of sleep compared to non-HCW (10). This study may have underestimated the psychological effect of the COVID-19 outbreak on HCW because it was conducted in the early phase of the outbreak. Another study from China reported that the prevalence of depression and anxiety was greater among HCW compared to non-HCW (11). However, HCW have been known to suffer greater psychological distress compared to non-HCW even before the COVID-19 outbreak, which may be due to more stressful working conditions (12). To ascertain the influence of the COVID-19 outbreak on the mental health of HCW, in comparison with non-HCW, a longitudinal study is needed to observe their mental health status before and after the outbreak of COVID-19. To date, no such study has been reported.

In this two-month (mid-March to mid-May 2020) longitudinal follow-up study of full-time employees conducted during the first wave of COVID-19 outbreak in Japan, we assessed changes in fear and worry of COVID-19, psychological distress, and physical symptoms among HCW and non-HCW.

## Methods

### Study design, participants, and procedure

In February 2019, we assessed a prospective online cohort of full-time employees, stratified by sex and 10-year age groups (N=4120), who had participated in a large digital marketing research company survey of community-dwelling people across Japan. Through an invitation e-mail from the company, we further invited these respondents to participate in a baseline online survey for this study administered on 19–22 March 2020 (T1). The questionnaire was closed once the target sample (up to 1500 participants) was obtained or the answer deadline came. For this T1 survey, a total of 1448 participated; response rate: 35.1% (13, 14).

The flowchart of participant recruitment is shown in the supplementary material (www.sjweh.fi/show_abstract.php?abstract_id=3922) figure 1. Respondents at T1 were more likely to be single and have no children compared to non-respondents. Differences in sex, age, or psychological distress scores between these two groups were statistically non-significant. After excluding unemployed respondents (N=27), we followed the remaining 1421 respondents for two months and surveyed them again on 22–26 May 2020 (T2). There were no missing responses in the questionnaire. Respondents who participated both in T1 and T2 surveys were included in further analyses.

At the time of the baseline survey (T1), the number of people infected by COVID-19 had just begun to increase rapidly in Japan with 1046 reported COVID-19 cases and 36 deaths. On 16 April, the Japanese Government declared a state of national emergency, which continued until 25 May. At the time of the follow-up survey (T2), 16 581 COVID-19 cases and 830 deaths had been reported in Japan (15).

The Research Ethics Committee of the Graduate School of Medicine/Faculty of Medicine at the University of Tokyo approved this study [No. 10856- (3)].

### Measurement variables

*Global fear and worry about COVID-19*. Global fear and worry about COVID-19 were assessed using a single item (13): “Do you feel anxiety about COVID-19?” Responses were rated on a 6-point Likert- scale ranging from 1=“No, not at all” to 6=“Yes, feel strongly.”

*Psychological distress and physical symptoms*. Psychological distress and physical symptoms in the last 30 days were measured with 18- and 11-item subscales, respectively, of the Brief Job Stress Questionnaire (BJSQ) (16, 17). Five subscales of psychological distress assessed lack of vigor, anger-irritability, fatigue, anxiety, and depression. The physical symptoms scale assessed various somatic symptoms (eg, loss of appetite, headache). All items were rated on a 4-point Likert scale from 1=“Never” to 4 =“Almost always”. The subscale scores were summed, with higher scores indicating greater distress or symptoms.

*Demographic variables*. Participants were asked about their occupations and whether they worked in healthcare facilities at T2. The response options included non-HCW (ie, general workers), HCW (ie, physicians, nurses/midwives), or other HCW (eg, pharmacists, clinical laboratory technicians) working in healthcare facilities, and HCW not working in healthcare settings (eg, public health centers, schools, or companies). We divided participants into two categories: all types of HCW (including both working and not working in healthcare settings) and non-HCW. We measured sex, age, marital status, having at least one child, educational attainment (≥16 years), residential area (living in or outside a prefecture in which the government had declared a COVID-19 special emergency) as covariates in statistical analyses. We also collected information on industry and organization size at T1.

### Statistical analysis

The mean change in scores of psychological distress and physical symptoms from T1 to T2 were compared between HCW and non-HCW (t-test for two independent groups). A general linear model with repeated measures using a first-order autoregressive (AR1) covariance matrix was used to estimate adjusted means of these outcomes between HCW and non-HCW, adjusting for the covariates (sex, age, marital status, having at least one child, education, and residential area). A differential change in the outcomes between the two groups was tested with a group×time interaction. Cohen’s d was adopted to calculate crude and adjusted effect size (ES). Statistical significance was set as P<0.0071 (=0.05/7) for a two-tailed statistical test applying the Bonferroni’s correction for the seven outcome variables. In addition, we conducted two sensitivity analyses. First, we conducted similar general linear model analyses with repeated measures using sampling weights to make the demographic distribution of sex, 10-year age groups, and occupation (HCW or non-HCW) of the sample comparable to that of the entire working population of Japan (see the supplementary table S1). Second, with dichotomizing fear and worry about COVID-19 into high (≥4) and low (≤3), prevalence of high fear and worry at T1 and T2 were compared between HCW and non-HCW (Fisher exact test). Multiple logistic regression was employed to estimate odds ratios (OR) and 95% confidence intervals (CI) of high fear and worry at T2 among HCW compared to non-HCW, adjusting for high fear and worry at T1 only and adjusting for high fear and worry at T2 and the covariates. SPSS 26.0 (IBM Corp, Armonk, NY, USA) Japanese version was used.

## Results

A total of 1032 participants (72.6%) completed the follow-up questionnaire at T2. Compared to those lost to follow-up at T2, the completers were statistically significantly more likely to be engaged in the education industry sector. Differences in sex, age, psychological distress, marital status, or having a child(ren) at baseline between the two groups were statistically non-significant (data available upon request). We excluded respondents who became unemployed (N=17). The final sample consisted of 111 HCW and 904 non-HCW. Table 1 shows the characteristics of each group of participants. Two-thirds of HCW were female, while the male-to-female ratio was close to 1 among non-HCW. Among HCW, most participants (55%) were healthcare workers other than physicians and nurses/midwives. HCW also included those who worked in non-clinical settings (28%).

**Table 1:** Characteristics of participants in the study (N=1015). [SD= standard deviation].

	Healthcare workers (N=111)		Non-healthcare workers (N=904)	
	
	N (%)	SD	N (%)	SD
Sex				
Male	39 (35.1)		472 (52.2)	
Female	72 (64.9)		432 (47.8)	
Age mean (years)	41.31	10.6	41.46	10.6
20–29	24 (21.6)		161 (17.8)	
30–39	35 (31.5)		246 (27.2)	
40–49	25 (22.5)		238 (26.3)	
50–59	24 (21.6)		242 (26.8)	
>60	3 (2.7)		17 (1.9)	
Marital status				
Single	54 (48.6)		437 (48.3)	
Married	57 (51.4)		467 (51.7)	
Child(ren)				
0	60 (54.1)		525 (58.1)	
≥1	51 (45.9)		379 (41.9)	
Education				
<16 years	39 (35.1)		414 (45.8)	
>16 years	72 (64.9)		490 (54.2)	
Residence				
Emergency prefectures ^a^	62 (55.9)		643 (71.1)	
Others	49 (44.1)		261 (28.9)	
Type of industry				
Manufacturing	8 (7.2)		238 (26.3)	
Medical and welfare	77 (69.4)		60 (6.6)	
Retail and wholesale business	6 (5.4)		99 (11.0)	
Finance, insurance, real estate	2 (1.8)		85 (9.4)	
Public service	11 (9.9)		69 (7.6)	
Information and technology services	- (-) ^b^		77 (8.5)	
Life-related services and entertainment	2 (1.8)		71 (7.9)	
Professional and technical services	2 (1.8)		53 (5.9)	
Transportation	1 (0.9)		45 (5.0)	
Education and learning support	2 (1.8)		43 (4.8)	
Construction	- (-) ^b^		36 (4.0)	
Eating/drinking, hotel business	- (-) ^b^		15 (1.7)	
Agriculture and industry	- (-) ^b^		5 (0.6)	
Others/unknown	- (-) ^b^		8 (0.9)	
Organization size (number of employees)				
≥1000	22 (19.8)		311 (34.4)	
300–999	28 (25.2)		150 (16.6)	
50–299	30 (27.0)		242 (26.8)	
<50	31 (27.9)		173 (19.1)	
Unknown	- (-) ^b^		28 (3.1)	
Healthcare worker details				
Physicians	4 (3.6)			
Nurses/midwives	15 (13.5)			
Other health care workers (eg, pharmacists, clinical laboratory technicians)	61 (55.0)			
Health care workers but not working in clinical settings	31 (27.9)			

a The Japanese Government has designated 13 prefectures (Tokyo, Osaka, Hyogo, Hokkaido, Ibaraki, Saitama, Chiba, Kanagawa, Ishikawa, Gifu, Aichi, Kyoto and Fukuoka) as specified prefectures for COVID-19.

b No cases.

The mean scores of fear and worry of COVID-19, psychological distress and its subscales, and physical symptoms were similar for HCW and non-HCW at T1 (table 2). Fatigue increased from T1 and T2 statistically significantly more among HCW than among non-HCW (P=0.005 <0.0071 with the Bonferroni’s correction). After adjusting for the covariates, the scores of total psychological distress and fatigue, anxiety, and depression subscales increased statistically significantly more among HCW compared to non-HCW (P= 0.002, 0.002, 0.003, and 0.006, respectively, all P<0.0071 with the Bonferroni’s correction). The global fear and worry of COVID-19 also increased more among HCW compared to non-HCW, although the difference was not statistically significant (P=0.049 >0.007). The estimated ES was greater for total psychological distress, as well as fatigue, anxiety, and depression (0.243–0.323 in Cohen’s d) than for fear and worry of COVID-19 (0.202).

**Table 2:** Fear and worry of COVID-19, psychological distress, and physical symptoms among healthcare and non-healthcare workers (HCW) (N=1015). [SD=standard deviation; SE=standard error].

Variables (possible range) ^a^	Crude				P-value	ES ^b^	Adjusted ^c^				P-value	ES ^b^
	
	HCW (N=111)		Non-HCW (N=904)				HCW (N=111)		Non-HCW (N=904)			
			
	T1 ^d^	T2 ^e^	T1 ^d^	T2 ^e^			T1 ^d^	T2 ^e^	T1 ^d^	T2 ^e^		
			
	Mean (SD)	Mean (SD)	Mean (SD)	Mean (SD)			Mean (SE)	Mean (SE)	Mean (SE)	Mean (SE)		
Global fear and worry of COVID-19 (1–6)	4.26 (1.39)	4.68 (1.17)	4.32 (1.18)	4.53 (1.19)	0.054	0.194	4.21 (0.11)	4.64 (0.11)	4.35 (0.04)	4.55 (0.04)	0.049	0.202
Psychological distress											
Total (18–72)	40.21 (10.92)	42.86 (11.66)	41.36 (11.69)	41.10 (11.06)	0.008	0.316	39.82 (1.11)	42.33 (1.07)	41.37 (0.43)	40.98 (0.41)	0.002 ^f^	0.316
Lack of vigor (3–12)	8.80 (2.46)	9.01 (2.36)	9.30 (2.40)	9.47 (2.30)	0.860	0.018	8.87 (0.23)	9.07 (0.22)	9.29 (0.08)	9.46 (0.08)	0.917	0.010
Anger-irritability (3–12)	6.74 (2.43)	6.98 (2.46)	6.89 (2.55)	6.70 (2.56)	0.073	0.180	6.58 (0.24)	6.83 (0.24)	6.91 (0.09)	6.70 (0.09)	0.066	0.189
Fatigue (3–12)	6.61 (2.44)	7.23 (2.53)	6.83 (2.68)	6.65 (2.59)	0.005 ^f^	0.327	6.49 (0.25)	7.10 (0.25)	6.86 (0.10)	6.68 (0.10)	0.002 ^f^	0.323
Anxiety (3–12)	6.59 (2.46)	7.26 (2.40)	6.58 (2.47)	6.53 (2.39)	0.008	0.316	6.52 (0.24)	7.17 (0.23)	6.55 (0.09)	6.50 (0.09)	0.003 ^f^	0.308
Depression (6–24)	11.47 (4.27)	12.38 (4.71)	11.75 (4.71)	11.75 (4.50)	0.019	0.236	11.37 (0.44)	12.22 (0.43)	11.79 (0.17)	11.70 (0.17)	0.006 ^f^	0.243
Physical symptom (11–44)	19.64 (6.05)	20.58 (6.66)	19.15 (6.35)	19.37 (6.52)	0.181	0.135	19.25 (0.60)	20.17 (0.62)	19.05 (0.23)	19.25 (0.24)	0.192	0.134

a Higher scores indicate greater global fear and worry of COVID-19, psychological distress, and physical symptoms.

b Adjusted effect size (ES) of the score changes between the two groups was calculated as Cohen’s d by dividing the crude and adjusted mean differences by SD of the crude pooled difference. Effect size was calculated for the changes in the scores from T1 to T2 in HCW compared to non-HCW. A positive ES value means that the scores increased more among HCWs than non-HCWs during the follow-up.

c Adjusted for sex, age, marital status, having at least one child, education, and residence (emergency prefectures or not).

d T1: 19-22 March 2020,

e T2: 22-26 May 2020.

f P<0.0071, significant with the Bonferroni’s correction for the difference of the change scores between health care workers and non- health care workers (t-test for the crude analyses; the group x time interaction by analysis of variance with repeated measures for the adjusted analyses).

Weighting the sample to make the demographic distribution comparable to the entire working population of Japan did not change the patterns observed in the non-weighted analyses described above in general (supplementary table S1). However, only fatigue increased statistically significantly more among HCW compared to non-HCW after adjusting for the covariates (P=0.003, P<0.0071 with the Bonferroni’s correction); otherwise, the difference was statistically non-significant. Prevalence of high fear and worry about COVID-19 increased from T1 to T2 both among HCW and non-HCW (supplementary table S2). Multiple logistic regression showed that HCW had a marginally statistically significantly higher OR of having high fear and worry at T2, after adjusting for the fear and worry at T1 and the covariates (adjusted OR 1.94, 95% CI 0.97–3.88, P=0.063).

### Post-hoc statistical power calculation

To statistically test (t-test) the T2-T1 difference in change scores of psychological difference between HCW (N=111) and non-HCW (N=904), with small effect size (0.2 in Cohen’s d), the statistical power was estimated as 0.634, with an alpha of 0.05.

## Discussion

Mean scores of fear and worry of COVID-19, psychological distress and its subscales, and physical symptoms at baseline were similar between HCW and non-HCW. However, most indicators deteriorated among HCW during the COVID-19 outbreak while they remained the same or even improved among non-HCW. In particular, after adjusting the covariates, psychological distress and its subscale of fatigue, anxiety, and depression increased statistically significantly more among HCW compared to non-HCW during the COVID-19 outbreak.

This study demonstrated that HCW were more likely than non-HCW to develop psychological distress during the COVID-19 outbreak. The findings are consistent with previous cross-sectional studies indicating that HCW showed a high prevalence of anxiety, depression, and other psychiatric symptoms (2, 6, 7, 11), suggesting that HCW experienced poor mental health during the COVID-19 outbreak. The patterns were similar but statistically significant only for fatigue when the sample weight was applied to make the demographic distribution of the present sample comparable to that of the entire working population of Japan. This is probably attributable to a reduced statistical power caused by applying the sample weighting to the limited size of the sample. This longitudinal study provides more compelling evidence that the mental health of HCW declined during the COVID-19 outbreak, not just because of their prior working conditions, and that the degree of deterioration was greater for HCW than for non-HCW.

Among non-HCW, psychological distress and physical symptoms did not change, although their fear of COVID-19 increased slightly. The finding is consistent with a previous longitudinal study conducted in China showing that stress, anxiety, and depression were stable in a general sample during a spike in the number of COVID-19 cases (18). However, those who experienced greater stress due to COVID-19 may have been more likely to be lost to follow-up, which could lead to an underestimation of the effect of COVID-19 on mental health among non-HCW. It is also plausible that some potentially vulnerable groups, such as people with chronic conditions or disabilities, may have experienced a deterioration of mental health during the COVID-19 outbreak (19), which could have been masked in the analysis of the entire non-HCW sample.

### Limitations

This study has several limitations. The number of HCW in this sample was small (N=111). HCW participating in an internet survey may have been biased. The distributions of sex and age in this sample represented the working population of Japan. The findings were similar when the sample was weighted to make the sex- and age-distributions comparable to that of the entire working population of Japan. However, the sample may still be biased in terms of some other demographic characteristics, such as education and income. These possibilities may limit the generalizability of the findings. Our sample of HCW included only a few physicians and nurses/midwives. Therefore, our findings may not be generalized to these professionals. Moreover, the sample consisted only of full-time employees. Furthermore, we did not ask HCW participants whether they were in charge of the treatment of COVID-19 patients, which is an important predictor of posttraumatic stress among HCW (2, 8, 20). We also did not ask about important characteristics of work settings, such as clinics or hospitals, where the HCW worked. Nevertheless, most HCW reported that they worked in large organizations, which could have been hospitals. The study sample also included HCW working in laboratory facilities or industries related to the manufacturing of medical equipment, which may have resulted in an underestimation of the effect of the COVID-19 outbreak. Furthermore, we did not consider whether the respondents tested positive for COVID-19 at any point in the study. Lastly, we did not measure posttraumatic stress as a unique indicator of mental health among HCW during the COVID-19 pandemic (2, 8, 9).

### Concluding remarks

The two-month longitudinal study revealed that psychological distress among HCW deteriorated during the early months of the COVID-19 outbreak in Japan. The study suggests that mental healthcare should be available to HCW working in diverse environments.

### Disclosure

Approval of the research protocol: This study was approved by the Research Ethics Committee of the Graduate School of Medicine/Faculty of Medicine at the University of Tokyo, No. 10856- (3).

Online informed consent was obtained from all participants with full disclosure and explanation of the purpose and procedures of this study. We explained that their participation was voluntary and that they could withdraw consent for any reason simply by not completing the questionnaire.

### Conflict of interest

The authors report no conflicts of interest.

### Funding

This work was supported by internal funds of the Department of Mental Health, Graduate School of Medicine at the University of Tokyo.

The sponsors had no role in the design and conduct of the study; collection, management, analysis, and interpretation of the data; preparation, review, or approval of the manuscript; or the decision to submit the manuscript for publication.

## Supplementary material

Supplementary material
