# Supplementary material for: The deterioration of mental health among healthcare workers during the COVID-19 outbreak: A population-based cohort study of workers in Japan
Source: Scand J Work Environ Health. 2020 Oct 30;46(6):639–44. doi: 10.5271/sjweh.3922 (PMC7737801; doi:10.5271/sjweh.3922)
Supplement: Supplementary material [file SJWEH-46-639-S001.pdf]

The deterioration of mental health among healthcare workers during the COVID-19 outbreak: A population-based cohort study of workers in Japan[1]

by Natsu Sasaki, MD, Reiko Kuroda, MD, PhD, Kanami Tsuno, PhD, Norito Kawakami, MD, PhD [2]

1. *Supplementary material*
2. *Correspondence to: Norito Kawakami, MD, Department of Mental Health, Graduate School of Medicine, The University of Tokyo, 7-3-1, Hongo, Bunkyo-ku, Tokyo, 113-0033, Tokyo, Japan. [E-mail: nkawakami@m.u-tokyo.ac.jp]*

Supplementary table S1

Fear and worry of COVID-19, psychological distress, and physical symptoms among health care and non-health care workers (N=1,015): a sensitivity analysis with weighting the sample to the distribution of sex, age groups, and occupation (health care workers or non-health care workers) of the whole working population of Japan. [a]

Variables [possible range] [b]	Health care workers (n=111)		Non-health care workers (n=904)		p	ES [d]	Health care workers (n=111)		Non-health care workers (n=904)		p	ES [d]
	Crude						Adjusted [c]					
	T1	T2	T1	T2			T1	T2	T1	T2		
	Mean (SD)	Mean (SD)	Mean (SD)	Mean (SD)			Adjusted mean (SE)	Adjusted mean (SE)	Adjusted mean (SE)	Adjusted mean (SE)		
Global fear and worry of COVID-19 [1 -6]	4.40 (1.45)	4.80 (1.22)	4.29 (1.16)	4.51 (1.17)	0.058	0.175	4.26 (0.11)	4.66 (0.11)	4.32 (0.04)	4.54 (0.04)	0.083	0.168
Psychological distress:												
Total [18 – 72]	40.73 (11.66)	42.72 (13.15)	41.16 (11.54)	40.96 (10.95)	0.010	0.237	40.11 (1.03)	42.11 (1.00)	41.25 (0.39)	41.06 (0.38)	0.014	0.239
Lack of vigor [3 – 12]	9.49 (2.26)	8.97 (2.59)	9.33 (2.37)	8.93 (2.63)	0.554	-0.055	8.89 (0.21)	8.86 (0.20)	9.36 (0.09)	9.50 (0.09)	0.427	-0.077
Anger-irritability [3 – 12]	6.80 (2.73)	6.97 (2.68)	6.83 (2.51)	6.66 (2.51)	0.124	0.143	6.60 (0.23)	6.84 (0.23)	6.86 (0.09)	6.68 (0.09)	0.073	0.174

Fatigue [3 – 12]	6.67 (2.63)	7.23 (2.83)	6.79 (2.63)	6.61 (2.57)	0.001*	0.297	6.51 (0.23)	7.05 (0.23)	6.82 (0.09)	6.64 (0.09)	0.003*	0.288
Anxiety [3 – 12]	6.52 (2.37)	7.26 (2.62)	6.54 (2.43)	6.52 (2.37)	0.012	0.232	6.68 (0.22)	7.20 (0.22)	6.55 (0.08)	6.53 (0.08)	0.015	0.236
Depression [6 – 24]	11.58 (4.57)	12.29 (5.30)	11.67 (4.65)	11.68 (4.44)	0.052	0.180	11.45 (0.41)	12.16 (0.41)	11.69 (0.16)	11.70 (0.15)	0.063	0.181
Physical symptom [11 – 44]	20.02 (6.63)	20.76 (7.11)	19.02 (6.27)	19.21 (6.40)	0.255	0.105	19.66 (0.56)	20.30 (0.58)	19.08 (0.21)	19.28 (0.22)	0.386	0.084

[a] Sampling weights were calculated as an inverse of a proportion ratio in the sample compared to the whole working population data from the Labor Forth Survey 2019 (N=51,760,000) for each of groups classified based on sex, age (10-year), and occupation (health care workers or non-health care workers). The population of HCWs was referred to the employees working in the medical and welfare industry. Weighted general linear model with repeated measures was employed to estimate P value for a difference in the change of an outcome variable from T1 to T2 between health care workers and non-health care workers.

[b] Higher scores indicate greater global fear and worry of COVID-19, psychological distress, and physical symptoms.

[c] Adjusted for sex, age, marital status, having at least one child, education, residence (emergency prefectures or not).

[d] Adjusted effect size (ES) of the score changes between the two groups was calculated as Cohen's d by dividing the crude and adjusted mean differences by SD of the crude pooled difference. Effect size was calculated for the changes in the scores from T1 to T2 in HCW compared to non-HCW. A positive ES value means that the scores increased more among HCWs than non-HCWs during the follow-up.

*p<0.0071 (=0.05/7), significant with the Bonferroni's correction for the difference of the change scores between health care workers and non- health care workers (t-test for the crude analyses; the group x time interaction by analysis of variance with repeated measures for the adjusted analyses).

Note: T1: 19-22 March 2020, T2: 22-26 May 2020. SD: standard deviation. SE: standard error.

Supplementary table S2.

Comparison of prevalences of global hear and worry about COVID-19 at T1 and T2 between health care workers and non- health care workers.

		Prevalence of global fear and worry about COVID-19 (High)				Odds ratio (OR) of high global fear and worry about COVID-19 at T2 among HCWs compared to non-HCWs controlling for the T1 value [b]					
		At baseline (T1)		At follow-up (T2)		Crude			Adjusted [c]		
	Total N	n	%	n	%	OR	95%CI	P	OR	95%CI	P
Health care workers	111	84	75.7	99	89.2	1.89	0.96 – 3.72	0.065	1.94	0.97-3.88	0.063
Non-health care workers	904	720	79.6	763	84.4	1.00			1.00		
P for difference [a]	1015		P=0.324		P=0.207						

[a] Fisher's exact test.

[b] Multiple logistic regression analysis.

[c] Adjusted for sex, age, marital status, having at least one child, education, and residence (emergency prefectures or not).

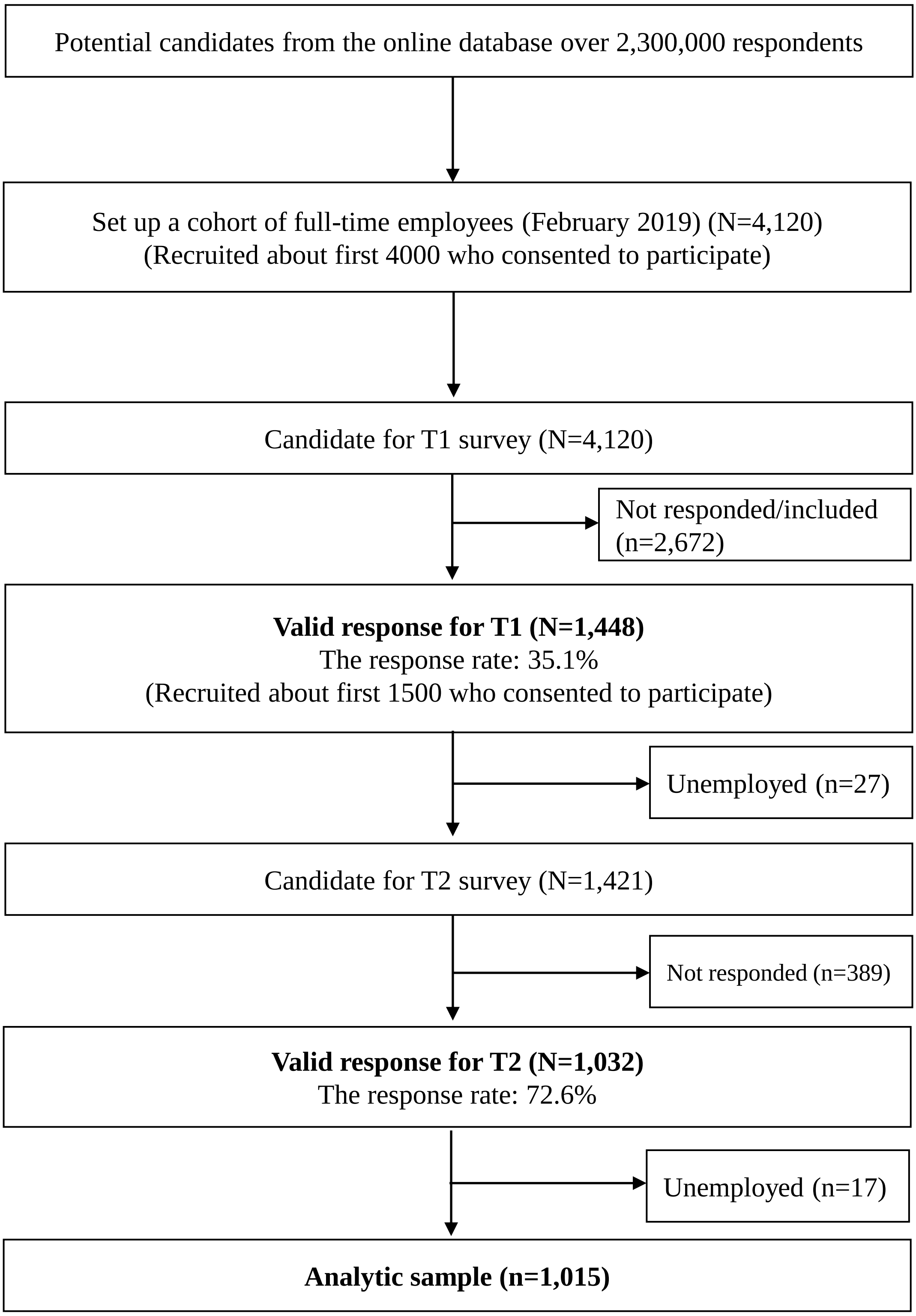

Supplemental figure 1. Flowchart of participant recruitment.
